# Utilizing Microbial Inoculants to Alleviate Continuous Cropping Obstacles: Insights into the Metabolites and Transcriptomic Responses of *Pinellia ternata*

**DOI:** 10.3390/metabo15030189

**Published:** 2025-03-11

**Authors:** Xinyu Wang, Mohammad Murtaza Alami, Shuqi Gong, Qinglin Cheng, Chaoqun Chen, Xinghui Li, Shumei Zhong, Zhigang He, Dilin Chen, Shengqiu Feng, Shenghu Chen, Shaohua Shu

**Affiliations:** 1 College of Plant Science & Technology, Huazhong Agricultural University, Wuhan 430070, China; wangxinyu111@webmail.hzau.edu.cn (X.W.);; 2 Sinopharm Zhonglian Pharmaceutical Co., Ltd., Wuhan 430070, China; 3 Hubei South Crown Chinese Herbs Science & Technology Co., Ltd., Qianjiang 433131, China; 4 Planting Industry Management Office, Department of Agriculture and Rural Affairs in Hubei Province, Wuhan 430070, China; 5 Fruit and Tea Industry Management Office, Department of Agriculture and Rural Affairs in Hubei Province, Wuhan 430070, China

**Keywords:** *Pinellia ternata*, continuous cropping, microbial inoculants, metabolomics, transcriptomics

## Abstract

*Pinellia ternata* (Thunb.) Breit is a widely used medicinal herb in Traditional Chinese Medicine (TCM). Still, its sustainable cultivation is threatened by continuous cropping obstacles, which disrupt soil ecosystems, reduce yield, and degrade quality. **Objectives:** This study explores the potential of microbial inoculants to mitigate these challenges through integrated metabolomic and transcriptomic analyses. **Methods**: Soil samples from fields with and without continuous cropping issues were used to compare the effects of microbial inoculants on the secondary metabolism and gene expression of *P. ternata*. **Results and Discussion:** Metabolomic profiling identified 20,969 metabolites, with significant changes in lipid-like molecules (22.2%), organic acids (9.1%), and phenylpropanoids (7.0%) under microbial treatment. Notable increases in phenylalanine and caffeic acid levels were observed in microbial-inoculated plants. Correspondingly, transcriptomic analysis revealed the upregulation of phenylalanine ammonia-lyase (PAL) and other stress-related genes, confirming the metabolic shifts. Clustering and machine learning analyses highlighted the critical roles of metabolites and genes in enhancing plant resilience. Microbial inoculants improved secondary metabolite production. **Implications:** These findings provide valuable insights into the mechanisms of microbial-plant interactions and establish a sustainable approach for cultivating *P. ternata*, addressing the challenges of continuous cropping while improving crop productivity and quality.

## 1. Introduction

*Pinellia ternata* (Thunb.) Breit, known as Banxia, is a well-known medicinal herb extensively used in Traditional Chinese Medicine (TCM). It is highly regarded for its therapeutic properties in treating dampness, phlegm, nausea, and abdominal distension. With over two millennia of use, *P. ternata* is significant in Chinese medicine and modern pharmaceutical formulations [1]. The increasing demand for this herb has spurred rapid growth in its cultivation, making it a valuable economic crop. However, the accelerated expansion of the *P. ternata* industry has brought significant challenges, particularly the issue of continuous cropping, which poses a critical threat to the sustainable cultivation of this medicinal plant.

Continuous cropping, also called continuous cropping obstacle, arises when the same crop or closely related species are cultivated in the same soil over multiple cycles. This practice disrupts the delicate balance of soil ecosystems, leading to a decline in plant resistance, increased susceptibility to pests and diseases, and reduced yield and quality [2,3,4]. The phenomenon becomes more pronounced under intensified, large-scale agricultural systems [5]. According to Pervaiz et al. (2020), continuous cropping obstacles are particularly severe when plants from the same family or closely related species are repeatedly grown in the same soil. Modern agricultural practices characterized by specialization and intensification have exacerbated these challenges, further hindering agricultural sustainability [6].

The effects of continuous cropping are not limited to *P. ternata*. Many crops, including fruits, vegetables, grains, and medicinal herbs, experience similar issues [2,3]. Among medicinal plants, nearly 70% of those relying on roots or rhizomes, such as *Rehmannia glutinosa*, *Panax notoginseng*, *Angelica sinensis*, and *Atractylodes macrocephala*, face severe continuous cropping problems [7]. This issue is compounded by increasing demands, limited land resources, and specific cultivation conditions, creating significant barriers to the sustainable development of Chinese herbal medicine agriculture.

Continuous cropping obstacles stem from a combination of factors, including the deterioration of soil physical and chemical properties, the accumulation of allelopathic or autotoxic substances, outbreaks of soil-borne diseases, and changes in soil microbial communities [4,6]. Among these, the alteration of soil microbial communities is considered the primary driver. Long-term monoculture leads to the accumulation of metabolic products such as organic acids, amino acids, sugars, and CO_2_ in the rhizosphere, creating a unique and often unfavorable soil environment [5,8,9]. These changes disrupt soil nutrient balance, alter pH, reduce soil organic matter, and impair soil particle and nutrient absorption characteristics, ultimately resulting in plant physiological and functional disorders [10]. For instance, the continuous cropping of carrots leads to an excessive accumulation of available phosphorus in the soil, while the continuous cropping of *Sapindus mukorossi* results in phosphorus surplus and potassium deficiency, causing severe nutrient imbalances [11]. High levels of phosphate in the soil strongly inhibit mycorrhizal colonization, which, in addition to water supply, greatly promotes mineral nutrition, nitrogen metabolism, and the enrichment of the amino acid pattern of plants [12].

The interaction between plants and soil microorganisms is crucial in maintaining soil health and crop productivity [10,13,14]. Plant root exudates, including sugars, amino acids, and organic acids, serve as vital nutrient sources for soil microorganisms, shaping the microbial community structure [15,16,17]. Root exudates also contain compounds such as flavonoids, sesquiterpenoid lactones, and terpenoids that facilitate underground chemical communication [18]. However, allelopathic substances with inhibitory effects, often referred to as autotoxic substances, can accumulate in the rhizosphere [19]. Phenolic acids, a key group of these substances, significantly impact microbial diversity and community structure. Their buildup in the soil selectively increases certain microbial populations while reducing beneficial bacteria that suppress pathogens [8,9,20]. For instance, in *Rehmannia glutinosa*, long-term monoculture leads to the accumulation of phenolic acids such as ferulic acid and vanillic acid, promoting the growth of pathogenic *Fusarium solani* and inhibiting beneficial *Pseudomonas* strains [21].

The challenges associated with continuous cropping are particularly evident in medicinal herbs like *P. ternata*, where the roots and rhizomes are the primary medicinal components. The repeated cultivation of *P. ternata* disrupts the rhizosphere’s microbial community, reduces beneficial microorganisms, and increases the prevalence of soil-borne diseases [22]. These changes affect crop yield and quality and necessitate the widespread use of chemical pesticides to manage diseases [23]. While effective in the short term, chemical inputs degrade soil health and pose significant environmental risks, further undermining the sustainable development of *P. ternata* cultivation. As such, there is an urgent need for alternative approaches that ensure the sustainability of the industry and the safety of medicinal products.

Recent studies highlight the complex interplay between plants and their rhizosphere microbial communities. Symbiotic relationships, such as those between nitrogen-fixing bacteria and legumes or mycorrhizal fungi and plants, enhance nutrient acquisition and promote plant growth [24,25]. Microorganisms also regulate plant metabolism and physiological processes by producing hormones or facilitating mucilage secretion. Conversely, plants influence microbial adaptation and evolution, fostering stable microbial communities that enhance nutrient absorption, suppress pathogens, and improve stress resistance [26,27]. However, continuous cropping disrupts these interactions, reducing microbial diversity and altering community structure, exacerbating soil-borne diseases and nutrient imbalances.

This study explores how microbial inoculants can mitigate the negative effects of continuous cropping on *P. ternata*. Specifically, the research investigates how prolonged cropping affects secondary metabolic processes and the transcriptomic responses in the roots of *P. ternata* and how these changes influence crop yield and quality. To achieve this, soils were selected from two conditions: one from seven years of continuous *P. ternata* cropping with cropping obstacles and another from soil without previous *P. ternata* planting. Three treatments were applied: microbial inoculants with cropping obstacles, a control with no inoculants and no obstacles, and a control with no inoculants but with obstacles. The study seeks to provide valuable scientific insights for developing sustainable cultivation strategies and maintaining soil ecosystem stability by elucidating the underlying mechanisms of continuous cropping stress. The findings will enhance the understanding of continuous cropping obstacles in medicinal herbs and offer practical solutions for improving the sustainability of Chinese herbal medicine agriculture.

## 2. Method and Materials

### 2.1. Experimental Design and Sampling

Soil samples were collected from fields with different cropping history durations: fields that had been continuously cropped and uncultivated soil. The continuously cropped fields had 106.6 mg/kg of available nitrogen, 31.8 mg/kg of available phosphorus, and 192.2 mg/kg of available potassium, while uncultivated soil had 106 mg/kg of available nitrogen, 44.2 mg/kg of available phosphorus, and 212.4 mg/kg of available potassium. These soils, which showed signs of continuous cropping issues, were investigated for the effects of microbial inoculants on *P. ternata*. The soil samples were air-dried in a shaded area, sieved to remove any remaining tubers, and thoroughly mixed to ensure uniformity. Lang Song is a compound microbial inoculant produced by Wuhan Langsong Biotechnology Co., Ltd. (Wuhan, China), mainly containing *Bacillus subtilis*, *Bacillus licheniformis*, and an algal growth stimulator with an effective viable count of >2 billion/g. The experimental treatments consisted of (i) inoculation with microbial inoculants and continuous cropping (JJ), (ii) no inoculation with microbial inoculants and no continuous cropping issues (N), and (iii) no inoculation with microbial inoculants but with continuous cropping issues (L). A total of three treatments were established. For each treatment, the fibrous roots of *P. ternata* were carefully washed with distilled water to remove any residual soil and then quickly blotted dry. The samples were placed in sterile tubes and immediately snap-frozen in liquid nitrogen before being stored at −80 °C for further analysis. Three biological replicates were taken for each treatment, with each replicate consisting of three pooled samples, which were then sent for metabolomic analysis.

### 2.2. Metabolites Profiling

The main steps of the experimental procedure include sample collection, metabolite extraction, quality control (QC) preparation, liquid chromatography–tandem mass spectrometry (LC-MS/MS) analysis, mass spectrometry analysis, and data processing. Raw data were converted to mzXML format using ProteoWizard (version 3) and processed with XCMS software (version 3.5.1) for peak alignment, retention time correction, and peak area extraction. The data obtained from XCMS were subjected to metabolite identification, data preprocessing, and an evaluation of experimental data quality. Following preprocessing, data analysis was performed, which included univariate statistical analysis, multivariate statistical analysis, differential metabolite screening, correlation analysis of differential metabolites, machine learning, ROC (Receiver Operating Characteristic) analysis, and KEGG (Kyoto Encyclopedia of Genes and Genomes) pathway analysis.

Metabolite identification was performed using a combination of a locally developed database and publicly available libraries. The identification process involved matching the retention time, molecular mass (with a mass error within <10 ppm), secondary fragmentation spectra, and collision energy of the metabolites in biological samples to those in the database. The identified metabolites were then manually re-checked and confirmed through rigorous secondary verification. Only metabolites identified with a confidence level of Level 2 or higher were considered valid. The identified metabolites were classified based on their chemical taxonomy, and the distribution of metabolites across different classes was statistically analyzed. The proportion of each metabolite class was visualized using a pie chart.

The OPLS-DA model generates variable importance values (Variable Importance for the Projection, VIP), which indicate how well each variable (feature peak) explains the X dataset and its association with the Y dataset. The sum of the squared VIP values equals the total number of variables in the model, with an average value of 1. When a variable has a VIP > 1, it indicates that the variable is significant, and it is commonly used as one of the criteria for identifying potential biomarkers. Thus, VIP values are used to assess the strength and explanatory power of metabolite expression patterns in discriminating between sample groups, helping identify metabolites with biological significance. A VIP > 1 and a *p*-value < 0.05 are typically used to select significantly different metabolites in metabolomics. Differential metabolite screening is performed in three modes: positive ion mode, negative ion mode, and combined positive and negative ion mode (with duplicate metabolites retained based on the higher VIP value).

Clustering analysis was used to assess the metabolic patterns of metabolites under different experimental conditions. Hierarchical clustering analysis was performed based on the relative values of metabolites under different experimental conditions, and the results were visualized as a heatmap. The heatmap represents a data matrix, visualizing differences between data points using a color gradient. This allows large differences to be emphasized while also highlighting smaller differences. Regions with different colors represent different clustering groups, where metabolites within the same group have similar metabolic patterns, potentially indicating similar functions or involvement in the same biological processes. Clustering metabolites with similar or related metabolic patterns makes it possible to infer their known or unknown biological functions. In this experiment, agglomerative hierarchical clustering was employed, where each object was initially considered a separate cluster, and these clusters were progressively merged into larger groups until the final grouping was reached. The relative quantitative values of the differential metabolites were scaled, and a hierarchical clustering dendrogram was generated using the Pheatmap package in R (version 4.4.3).

### 2.3. RNA Sequencing

This study enriched mRNA from total RNA using Oligo(dT) magnetic beads to capture polyadenylated transcripts. RNA fragmentation was achieved through ion-based shearing, producing approximately 300 base pairs (bp) fragments. The choice of 300 bp as the fragment length was based on the following considerations: shorter fragments would result in an overrepresentation of adapter sequences, thus reducing the proportion of valid data. In comparison, longer fragments could hinder efficient cluster formation during sequencing. cDNA synthesis was carried out using RNA as a template, with a six-base random primer and reverse transcriptase for the first-strand synthesis. The second strand of cDNA was then synthesized using the first strand as a template. Following cDNA synthesis, PCR constructed and amplified the library to enrich for desired fragment sizes. Library selection was performed based on fragment size, with a target size of 450 bp. The quality of the library was assessed using an Agilent 2100 Bioanalyzer, and both the library’s total concentration and effective concentration were measured. Each was labeled with a unique index sequence to distinguish samples; the libraries were then pooled proportionally to their required sequencing depth. The pooled libraries were diluted to 2 nM and denatured by alkali treatment to generate single-stranded libraries. After RNA extraction, purification, and library construction, the samples were subjected to second-generation sequencing (Next-Generation Sequencing, NGS) on the Illumina platform, utilizing paired-end (PE) sequencing technology. The raw sequencing data in FASTQ format were first subjected to quality filtering. Reads containing adapter sequences, those shorter than 50 bp, or those with an average quality score below Q20 were removed. The remaining high-quality reads were then assembled de novo to generate transcript sequences. These transcripts were clustered, and the longest transcript from each cluster was selected as the Unigene. Subsequently, functional annotation of the Unigenes was performed using various databases, including GO (Gene Ontology), KEGG (Kyoto Encyclopedia of Genes and Genomes), eggNOG (Evolutionary Genealogy of Genes: Non-supervised Orthologous Groups), SwissProt, and Pfam. Open Reading Frame (ORF) prediction and Simple Sequence Repeat (SSR) prediction were also carried out. Filtered reads were aligned to the Unigenes, and the corresponding read count for each Unigene was determined. Based on these counts, differential expression analysis, enrichment analysis, and analyses of cSNPs (coding Single-Nucleotide Polymorphisms) and InDels (insertions and deletions) were performed.

## 3. Results

### 3.1. Microbial Inoculants and Continuous Cropping Changed the Metabolite Profiles

**Figure 1:**
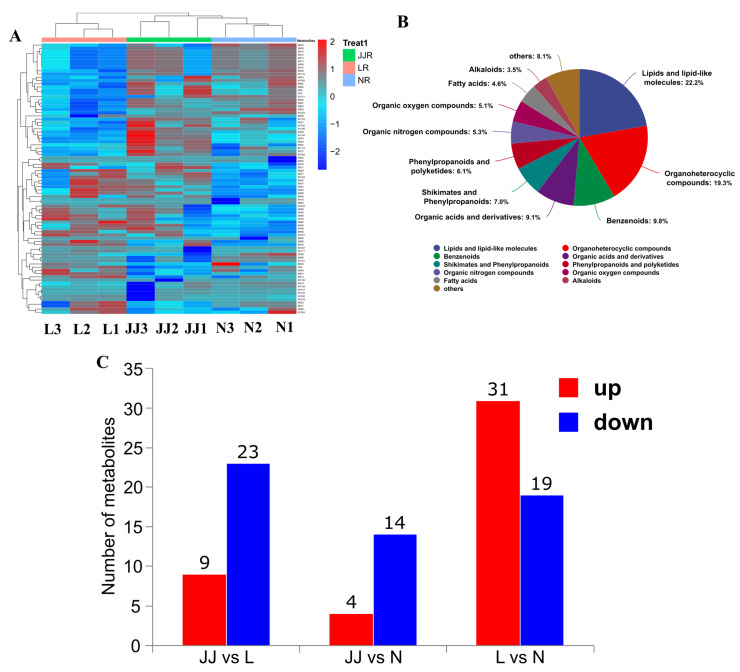
Metabolite identification classification. (**A**): Heatmap of the top 50 identified metabolite’s relative content. (**B**): The colored sections in the figure represent different chemical classification categories. The percentages indicate the proportion of metabolites within each classification relative to the number of identified metabolites. The chart primarily displays the top 10 largest classification categories. (**C**): Differential metabolites. The *x*-axis represents the comparison groups, and the *y*-axis shows the number of differential metabolites. JJ: Inoculation with microbial agents and continuous cropping. L: No inoculation with microbial agents but with continuous cropping issues. N: No inoculation with microbial agents and no continuous cropping issues.

A total of 1225 metabolites were successfully identified during the analysis (Figure 1A). This comprehensive identification was achieved through the combination of LC-MS/MS and advanced data processing techniques. Figure 1B primarily displays the top 10 largest classification categories, which are as follows: lipids and lipid-like molecules (22.2%), organic–heterocyclic compounds (19.3%), aromatic compounds (9.8%), organic acids and their derivatives (9.1%), shikimic acid and phenylpropanoids (7.0%), phenylpropanoids and polyphenolic compounds (6.1%), organic nitrogen compounds (5.3%), organic oxygen compounds (5.1%), fatty acids (4.6%), and alkaloids (3.5%). These categories represent the diverse metabolic pathways and biological functions involved in the studied samples. The distribution of metabolites across these categories reflects the complexity and biochemical diversity of the biological system under investigation.

As shown in Figure 1C, the continuous cropping group L and the control group N have the largest number of differential metabolites. In this comparison, there are 31 upregulated compounds and 19 downregulated compounds. This differential analysis highlights the metabolic changes induced by continuous cropping, shedding light on specific metabolic shifts in response to continuous cropping. On the other hand, the comparison between microbial inoculants and continuous cropping (JJ vs. L) showed that 23 metabolites are upregulated and 9 metabolites are downregulated. Finally, the comparison between the microbial inoculant and the control (JJ vs. N) showed the lowest metabolite regulation, with 4 upregulated and 14 downregulated. The upregulated compounds suggest potential biomarkers or pathways of interest for further exploration, while the downregulated metabolites could provide insight into processes that are suppressed or altered under experimental conditions.

#### 3.1.1. Differential Metabolite Clustering Analysis

**Figure 2:**
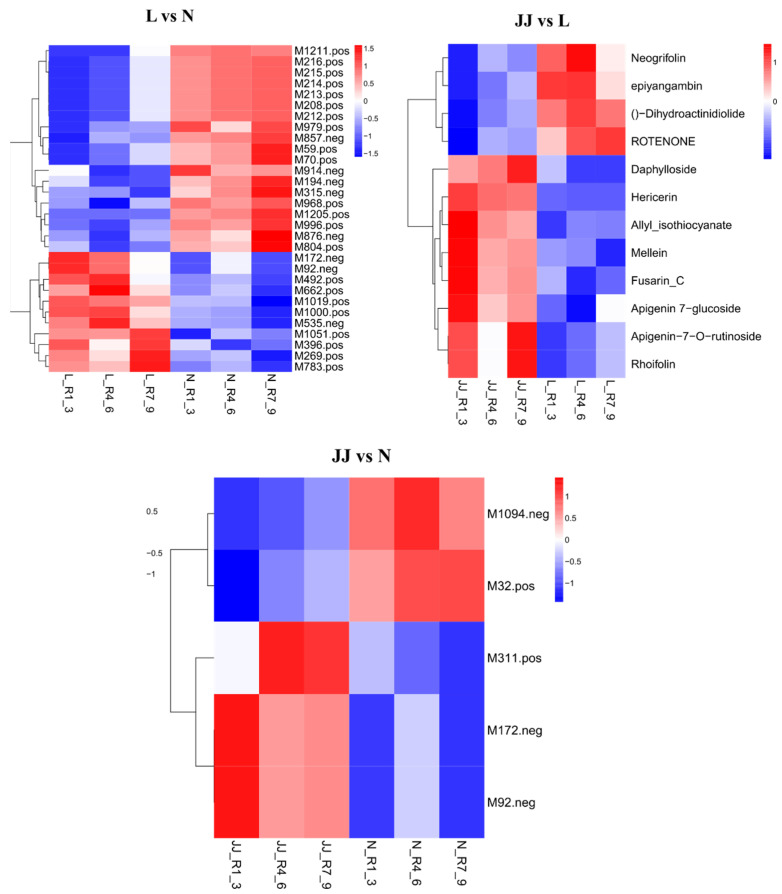
Clustering heatmap of JJR vs. LR. Some compound names are too long to fit in the figure; a corresponding table is provided. JJ: Inoculation with microbial agents and continuous cropping. L: No inoculation with microbial agents but with continuous cropping issues. N: No inoculation with microbial agents and no continuous cropping issues.

The heatmaps illustrate a differential metabolite clustering analysis across three experimental comparisons: L vs. N, JJ vs. L, and JJ vs. N (Figure 2). Each heatmap shows the relative importance of various metabolites in each comparison, using a color scale where red indicates high positive importance and blue signifies high negative importance. In the L vs. N comparison, the heatmap displays a complex pattern of metabolite importance, with notable metabolites such as M211_pos and M15_pos showing significant positive importance while M1986_pos and M173_neg show negative importance. The variation across the heatmap suggests a diverse impact of these conditions on metabolite levels, with specific metabolites being upregulated or downregulated significantly. In the JJ vs. L comparison, this heatmap is densely populated with red, indicating a prevalence of metabolites with high positive importance, such as Necroflorin and Dihydroactinidiolide, suggesting a strong upregulation or a key role of these metabolites under the conditions compared. Conversely, a few metabolites like Allyl isothiocyanate and ApiGenin 7-O-rutinoside are shown as less important or negatively important. In the JJ vs. N comparison, a smaller selection of metabolites is analyzed, with distinct clustering visible. Metabolites like M311_pos and M32_pos show high positive importance, whereas M1094_neg shows substantial negative importance, indicating that these metabolites are crucial in differentiating the JJ group from the N group regarding their metabolic profiles.

These analyses provide a detailed view into how experimental conditions or treatments influence metabolite dynamics within biological systems, highlighting specific metabolites that may play crucial roles in physiological or pathological processes associated with each experimental condition. This information could be invaluable for understanding metabolic pathways and investigating their biological significance.

#### 3.1.2. Differential Metabolite Machine Learning Analysis

**Figure 3:**
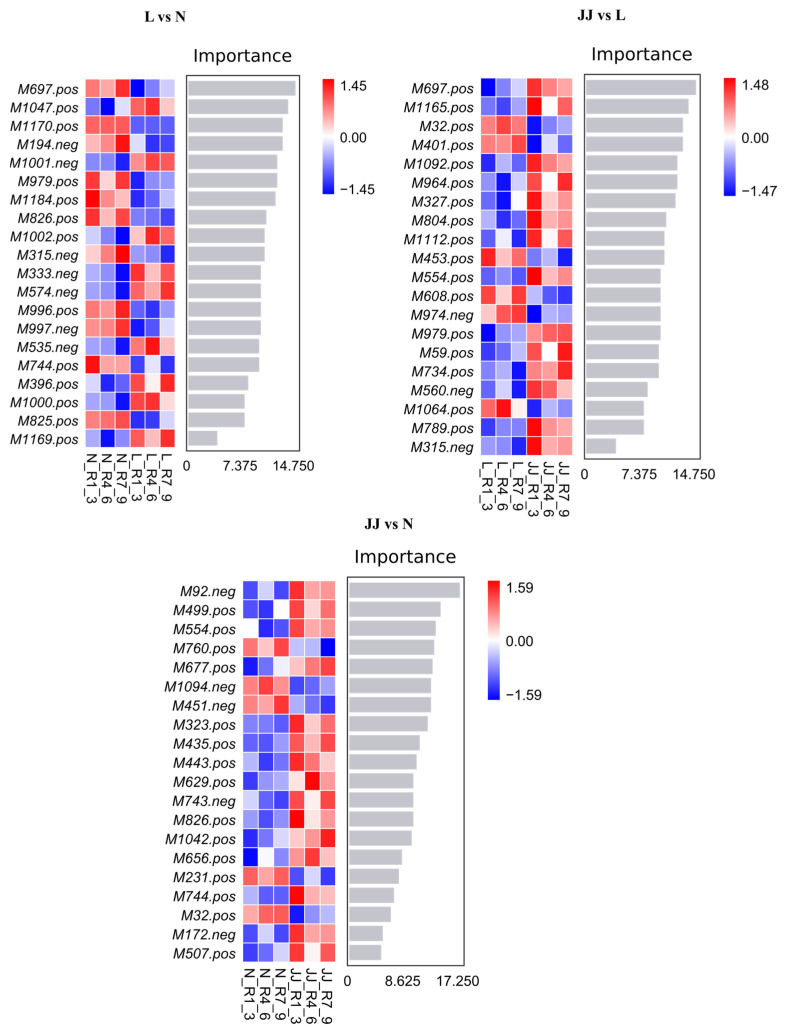
Random forest plot. The bar chart shows the importance scores of metabolites for the classifier model on the *x*-axis, with metabolite names listed on the *y*-axis. The heatmap displays the abundance distribution of these metabolites across different samples/groups. From top to bottom, the importance of metabolites for the model decreases. The metabolites with higher importance can be considered marker metabolites for the inter-group differences. JJ: Inoculation with microbial agents and continuous cropping. L: No inoculation with microbial agents but with continuous cropping issues. N: No inoculation with microbial agents and no continuous cropping issues.

Compared to a single decision tree model, the random forest algorithm leverages both sample and feature randomness to reduce the model’s variance, thereby enhancing the stability and generalizability of the model (Figure 3). The random forest analysis used the recursive feature elimination (RFE) algorithm for feature selection. The importance of each metabolite in the random forest model was measured by calculating the classification error (classif.ce). Iterations were performed at specific subset sizes, with the least important features being progressively removed to select the most significant features until the desired number of features was reached.

The heatmaps illustrate distinct patterns of feature importance across three experimental comparisons: L vs. N, JJ vs. L, and JJ vs. N. In the L vs. N comparison, features such as M7697_pos and M1704_neg display strong importance, indicating their significant role in differentiating these conditions. The JJ vs. L heatmap reveals a similar variation in feature importance, with several features consistently showing strong positive or negative associations, such as M7697_pos and M402_pos, highlighting their potential critical impact on the biological or pathological distinctions between these groups. Finally, in the JJ vs. N comparison, features like M292_neg and M4995_pos are important, suggesting that they are key in understanding the differences induced by the experimental conditions or treatments in groups JJ compared to N. These heatmaps collectively provide valuable insights into the specific genes or proteins that may be crucial in the biological pathways affected by the experimental conditions studied.

### 3.2. Microbial Inoculants and Continuous Cropping Changed the Transcripts

For transcriptome sequencing projects without a reference genome, we employed Trinity software (Trinity-v2.11.0) to assemble clean reads into transcripts for subsequent analysis. Trinity is a de novo assembly tool that uses the De Bruijn Graph (DBG) splicing method to process high-quality sequences. Once the assembly is complete, a transcript sequence file in FASTA format is generated. The longest transcript for each gene, referred to as a unigene, is extracted as the representative sequence for that gene (Table S2).

The Unigenes were functionally annotated using several databases, including NR (NCBI non-redundant protein sequences), GO (Gene Ontology), KEGG (Kyoto Encyclopedia of Genes and Genomes), eggNOG (Evolutionary Genealogy of Genes: Non-supervised Orthologous Groups), Swiss-Prot, and Pfam (Table S3). KO and Pathway annotations were primarily performed using the KOBAS annotation system, which is available at http://kobas.cbi.pku.edu.cn/ (accessed on 1 November 2024). The species category (e.g., animals, plants) was selected for the gene set. After KO annotation, the KO terms were mapped to the corresponding KEGG pathway. The statistical results of the KEGG pathway annotation are shown in Figure S1. The most annotated metabolic pathway in the KEGG annotation analysis was the signal transduction pathway, followed by carbohydrate metabolism, translation processes, and key biological processes such as substance transport and catabolism.

**Figure 4:**
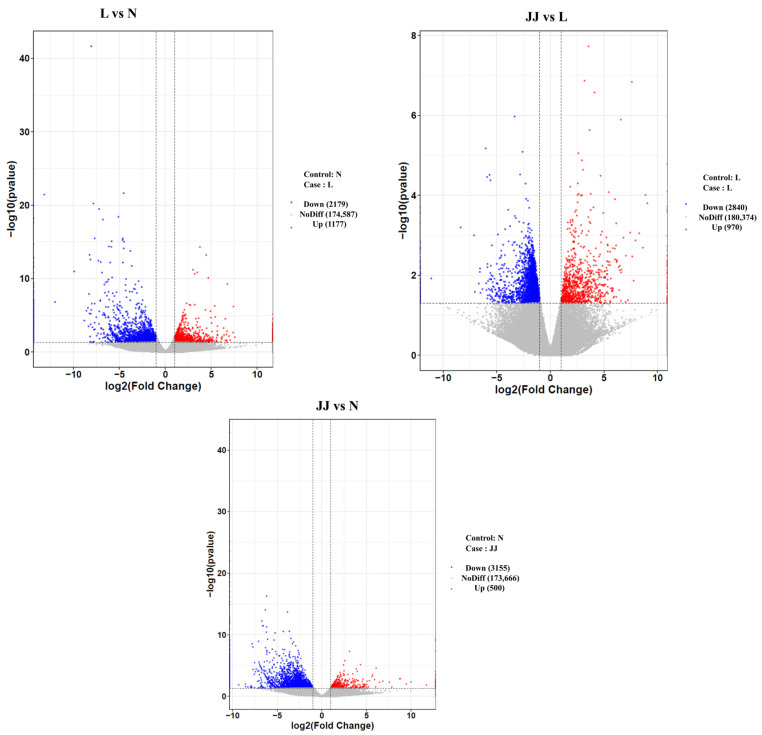
Volcano plot of differentially expressed genes (L vs. JJ). The *x*-axis represents log2FoldChange, and the *y*-axis represents −log10(*p*-value). The two vertical dashed lines indicate the 2-fold expression change threshold, and the horizontal dashed line represents the *p*-value = 0.05 threshold. Red dots represent upregulated genes in the group, blue dots represent downregulated genes, and gray dots represent genes with non-significant differential expression. JJ: Inoculation with microbial agents and continuous cropping. L: No inoculation with microbial agents but with continuous cropping issues. N: No inoculation with microbial agents and no continuous cropping issues.

We used DESeq for differential gene expression analysis. The criteria for selecting differentially expressed genes were fold change |log2FoldChange| > 1 and significance *p*-value < 0.05. The statistical results for differentially expressed genes between groups are shown in Figure 4 and Figure S2. A volcano plot of differentially expressed genes was created using the ggplot2 package in R, as shown in Figure 4. The volcano plot displays the gene distribution, fold change, and significance results. Under normal conditions, the plot should show a roughly symmetrical distribution of differentially expressed genes, with the left side representing downregulated genes in the case group compared to the control group and the right side representing upregulated genes in the case group compared to the control group.

The patterns of gene expression and their significance suggest differential impacts of the conditions or treatments associated with each group. Group JJ shows a distinctive response in both comparisons, which may indicate a unique or stronger treatment or condition effect compared to group L. The differences in gene expression highlight the specific genes that might be involved in response mechanisms and suggest potential pathways that could be affected by the experimental conditions. These results could be crucial for further investigations into the biological processes influenced by the conditions tested, potentially leading to new insights into cellular mechanisms or the development of targeted therapies or diagnostics based on the affected pathways and genes.

**Figure 5:**
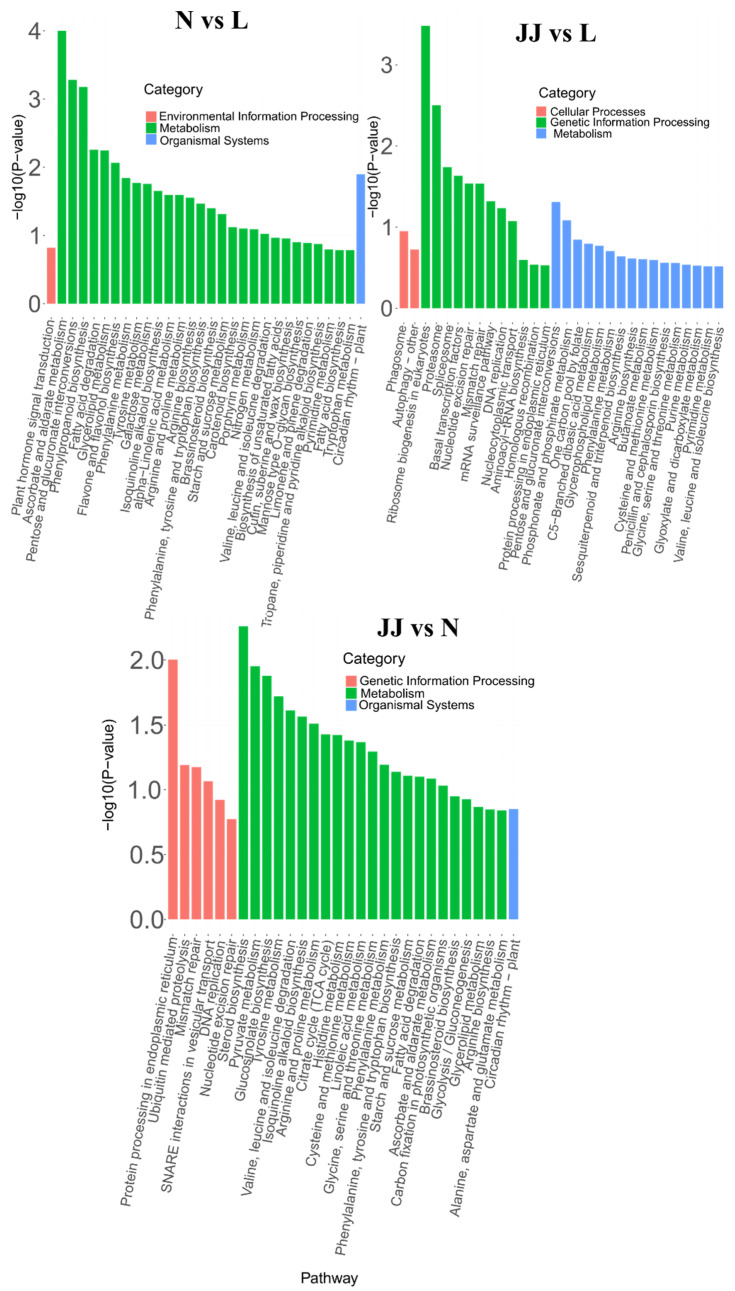
KEGG pathway enrichment results (bar plot) (N vs. L). The *x*-axis represents the pathway names, and the *y*-axis represents the −log10 (*p*-value) for the enrichment of each pathway. JJ: Inoculation with microbial agents and continuous cropping. L: No inoculation with microbial agents but with continuous cropping issues. N: No inoculation with microbial agents and no continuous cropping issues.

Based on the KEGG enrichment analysis results of differentially expressed genes, the top 30 pathways with the smallest *p*-value (i.e., the most significantly enriched) were selected for presentation. The results are shown in Figure 5. The enrichment results for N vs. L and N vs. JJ show that most differentially expressed genes are enriched in metabolic pathways, indicating that these genes play an important role in cellular metabolic balance, energy metabolism, or metabolic adaptive responses.

In N vs. L comparison, the pathways showing significant differences are predominantly in the ‘Environmental Information Processing’ category, with some involvement in ‘Metabolism’ and ‘Organismal Systems’. The most important pathway in this comparison is ‘Vascular Smooth Muscle Contraction’, suggesting a strong differential regulation of this pathway between groups N and L. The JJ vs. L comparison shows a mix of significant pathways across ‘Cellular Processes’, ‘Genetic Information Processing’, and ‘Metabolism’. ‘Protein Export’ is the most important pathway, highlighting a potentially unique aspect of cellular function that differs between the JJ and L conditions. In the JJ vs. N comparison, similarly to the JJ vs. L comparison, ‘Protein Export’ is the most significantly impacted pathway, indicating that this pathway consistently differs between JJ and both N and L groups. The result also shows a broad range of pathways affected, mostly within ‘Metabolism’, suggesting that metabolic processes are particularly distinct in the JJ group compared to N and L.

Overall, these findings suggest that the experimental conditions or treatments associated with groups N, L, and JJ significantly impact specific cellular pathways. The repeated significance of ‘Protein Export’ in the JJ comparisons may point to this pathway’s critical role under the conditions or treatments experienced by the JJ group. This could be of particular interest for further investigation, potentially in the context of disease mechanisms, treatment responses, or cellular function regulation.

### 3.3. Microbial Inoculants and Continuous Cropping Changed the Enzymes and Genes Involved in the Phenylpropanoid Biosynthesis Pathways

**Figure 6:**
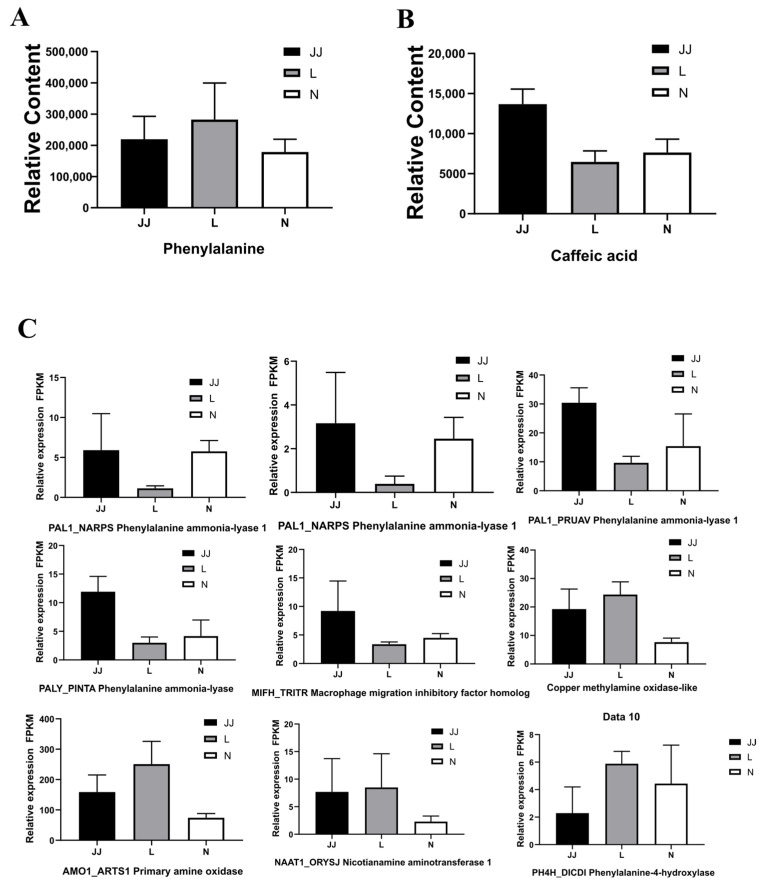
Phenylpropanoid biosynthesis pathways’ enzymes and gene expression. (**A**): the relative content of phenylalanine. (**B**): The relative content of caffeic acid. (**C**): The relative expression (FPKM) of genes involved in phenylpropanoid biosynthesis pathways. JJ: Inoculation with microbial agents and continuous cropping. L: No inoculation with microbial agents but with continuous cropping issues. N: No inoculation with microbial agents and no continuous cropping issues. Three biological replicates were used for each treatment. The relative expression was analyzed by calculating the mean values from these replicates.

In our untargeted metabolomic analysis, we assessed the relative content of phenylalanine and caffeic acid across three treatments: continuous cropping soil (L), microbial inoculant treatment (JJ), and a control (N). The relative content of phenylalanine (Figure 6A) varied significantly among the treatments, with the highest levels observed in the continuous cropping soil (L) and the lowest in the control (N). The microbial inoculant treatment (JJ) exhibited moderate levels of phenylalanine. In contrast, for caffeic acid (Figure 6B), the microbial inoculant treatment (JJ) displayed the highest levels, while the continuous cropping soil (L) and control (N) had significantly lower amounts, with the control showing the least. This differential profile suggests distinct regulatory or metabolic pathways affecting phenylpropanoid synthesis among the treatments.

Transcriptomic data (Figure 6C) elucidated the relative expression levels of several genes involved in the phenylalanine biosynthesis pathway. The expression levels of phenylalanine ammonia-lyase 1 (PAL1), the pivotal enzyme initiating the phenylpropanoid pathway, were elevated in the microbial inoculant treatment (JJ) compared to continuous cropping soil (L) and the control (N). This pattern correlates with the observed phenylalanine content, indicating a transcriptional regulation mechanism that aligns with metabolite levels. Further analysis showed the increased expression of other pathway-related enzymes in the microbial inoculant treatment (JJ) and continuous cropping soil (L) compared to the control (N). Notably, genes such as the macrophage migration inhibitory factor homolog (MIFH_TRITR) and copper methylamine oxidase-like gene exhibited higher expression in treatments JJ and L.

Additionally, genes encoding primary amine oxidase (AMO1_ARTS1) and nicotianamine aminotransferase 1 (NAAT1_ORYSJ) also showed elevated expression in these treatments, consistent with the enhanced activity of pathway enzymes and the increased phenylalanine levels observed. The correlation between metabolomic and transcriptomic data underscores a robust genetic regulation of the phenylalanine biosynthesis pathway, which differentially affects the production of related metabolites across the treatments. These findings illuminate the complex interplay between gene expression and metabolic processes, explaining the phenotypic differences observed among the treatments.

## 4. Discussion

The demand for *P. ternata*, an important medicinal in Traditional Chinese Medicine (TCM), has led to its increased cultivation, posing significant agricultural and ecological challenges [22,28,29]. This study highlights the critical role of microbial inoculants in overcoming the challenges of continuous cropping in *P. ternata* by leveraging a comprehensive omics approach. The findings demonstrate that microbial inoculants enhance nutrient assimilation and drive significant shifts in metabolic and transcriptomic profiles, particularly by elevating phenylpropanoids and other key secondary metabolites. Microbial inoculants enable *P. ternata* to thrive under adverse conditions by regulating metabolic balance and activating stress-adaptive pathways. These results establish microbial inoculants as a powerful, eco-friendly tool for promoting plant health, improving productivity, and mitigating the ecological consequences of continuous cropping [30,31,32]. This study provides a promising framework for integrating natural biological processes into sustainable agricultural systems.

Shikimic acid and phenylpropanoids, terpenoids, lignans, neolignans, and related compounds; and organic acids and their derivatives may be used for their insect-repellent, antimicrobial, and antifungal properties. These metabolites could be associated with continuous cropping problems in *P. ternate*. The metabolomic results revealed a substantial increase in key metabolites, with increasing phenylalanine content and caffeic acid in treated plants compared to controls. Moreover, secondary metabolites, including lipids and heterocyclic compounds, exhibited an overall increase, indicating a broad activation of metabolic pathways essential for stress defense in this study, which is inconsistent with Kumari et al. 2024’s finding that microbial inoculants significantly enhance the synthesis of secondary metabolites in medicinal plants by acting as stimuli that trigger metabolic modifications, thereby improving plant resilience against both biotic and abiotic stressors [33]. In chamomile plants, Schmidt et al. 2014 also reported that certain bacterial inoculants, specifically *Bacillus subtilis* Co1-6 and *Paenibacillus polymyxa* Mc5Re-14, significantly influence the community structure of the microbiome in chamomile plants and enhance the production of the bioactive secondary metabolite apigenin-7-O-glucoside [34]. The observed increases in phenylalanine and caffeic acid levels reflect a broader reprogramming of the metabolic network in *P. ternata*, driven by microbial inoculants and their influence under continuous cropping pressures. These shifts represent a systemic enhancement of secondary metabolite production, including lipids, heterocyclic compounds, and organic acids, critical for the plant’s stress response and adaptive capacity [35,36]. The targeted elevation of specific metabolites, such as shikimic acid and phenylpropanoids, highlights a strategic activation of key pathways that underpin the plant’s ability to adapt to challenging growth conditions [37,38]. These findings solidify the role of microbial inoculants in bolstering plant defenses against biotic and abiotic stressors, offering a promising avenue for enhancing resilience in agricultural systems.

Microbial inoculants demonstrated the ability to activate specific metabolic pathways that enhance plant defenses and growth under the challenges of continuous cropping, as evidenced by this study on *P. ternata*. The significant increase in lipid-like molecules and organic acids observed in treated plants suggests that microbial inoculants strengthen membrane integrity and optimize energy metabolism, both of which are critical for maintaining plant health under environmental stress [32,33,39]. These metabolic improvements are particularly valuable for plants subjected to repeated cropping cycles, where nutrient depletion and pathogen accumulation are persistent issues [31]. Furthermore, the variability in metabolic responses identified through OPLS-DA analysis highlights the selective capacity of microbial inoculants to enhance beneficial pathways while mitigating adverse conditions [33]. This tailored modulation of plant metabolism positions microbial inoculants as a versatile and sustainable tool for improving crop performance across diverse agricultural environments.

The transcriptomic analysis presented in this study provides valuable insights into how microbial inoculants and continuous cropping impact the gene expression profiles of *P. ternata*, particularly concerning stress responses and metabolic pathways. Transcriptomic data revealed that continuous cropping alone caused significant changes in gene expression, particularly genes involved in stress response, oxidative damage, and pathogen resistance, consistent with previous studies indicating that monoculture leads to plant stress [40]. In particular, the expression of phenylpropanoid pathway genes was strongly impacted, with PAL1, a key enzyme in this pathway, showing increased expression in the microbial inoculant plus continuous cropping and continuous cropping groups compared to the control. This upregulation corresponds to higher levels of phenylalanine, a precursor to various important secondary metabolites, suggesting a transcriptional regulatory mechanism that links stress response and metabolic adaptation [41]. In contrast, microbial inoculants in the JJ group significantly altered gene expression, particularly for genes related to phenylpropanoid biosynthesis and protein export pathways. The DESeq analysis highlighted the differences in gene expression between the groups, with notable upregulation in stress-related genes and those involved in nutrient acquisition in the JJ group. These findings align with the observation that microbial inoculants alleviate some of the negative impacts of continuous cropping by improving nutrient availability and supporting plant–microbe symbiosis [42]. This is further reflected in the enrichment of metabolic pathways such as protein export, a process essential for cellular function under stress conditions.

The volcano plot of DEGs further illustrated the significant differences between groups, particularly in the JJ group, which exhibited a distinct gene expression profile. The KEGG enrichment analysis indicated that metabolic pathways, particularly those related to cellular information processing and metabolism, were enriched in the JJ group, suggesting that microbial inoculants may enhance cellular metabolism, including energy production and substance transport. This contrasts with the L group, which showed a predominance of stress-related pathways like oxidative stress and cell wall modification, highlighting the detrimental effects of continuous cropping without microbial support. The differential expression of phenylpropanoid biosynthesis genes, such as PAL1 and other enzymes like the macrophage migration inhibitory factor homolog (MIFH_TRITR), copper methylamine oxidase-like enzyme, and primary amine oxidase (AMO1_ARTS1), was significantly higher in both the JJ and L groups compared to the N group. This pattern suggests that the microbial inoculants might be promoting phenylpropanoid synthesis by stimulating the transcription of relevant biosynthetic genes, which in turn may influence the production of secondary metabolites like phenylalanine and caffeic acid (Figure 6). The observed metabolic shifts imply that microbial inoculants could potentially optimize the metabolic networks in *P. ternata* by improving the plant’s ability to adapt to stress, particularly under conditions of continuous cropping.

Interestingly, the analysis of caffeic acid levels revealed that the JJ group, with microbial inoculants, had the highest content, supporting the hypothesis that microbial inoculants may directly or indirectly enhance the biosynthesis of key metabolites involved in plant defense mechanisms and secondary metabolism [10,29,42]. This suggests that microbial inoculants might influence enzyme activity in ways that foster more efficient metabolic pathways, thus improving plant health and resistance to the challenges posed by continuous cropping. Moreover, the study also highlighted the differential expression of genes in the protein export pathway, particularly under the JJ treatment, where protein export was significantly altered. This suggests that microbial inoculants might have a profound impact on protein synthesis and trafficking, which is vital for plant growth and stress adaptation. The pathway’s consistent involvement across multiple comparisons (e.g., JJ vs. N and JJ vs. L) suggests that it could play a critical role in mediating the beneficial effects of microbial inoculants.

## 5. Conclusions

This study underscores the transformative potential of microbial inoculants in mitigating continuous cropping stress in *P. ternata* through a comprehensive integration of metabolomic and transcriptomic approaches. By identifying significant increases in phenylalanine and caffeic acid, alongside the upregulation of phenylpropanoid biosynthesis genes such as phenylalanine ammonia-lyase (PAL1), this research establishes a robust mechanistic link between microbial treatments and enhanced plant resilience. These findings deepen our understanding of the biochemical and genetic pathways driving stress adaptation and position microbial inoculants as a sustainable and eco-friendly solution to agricultural challenges. Importantly, the synergy between enhanced metabolite production and gene expression illustrates how microbial inoculants can restore soil ecosystems, promote plant productivity, and reduce reliance on chemical inputs. This work contributes to the broader field of sustainable agriculture by offering a targeted, molecular-level framework for addressing continuous cropping obstacles in medicinal plant cultivation, paving the way for more resilient and sustainable cropping systems.

## Data Availability

The datasets generated and/or analyzed during the current study are uploaded to the SRA repository of NCBI with the SRA accessions, which are listed in Table S5.

## Supplementary Materials

The following supporting information can be downloaded at https://www.mdpi.com/article/10.3390/metabo15030189/s1. Figure S1. KEGG annotation statistics. Statistical results of the KEGG pathway annotation, showing the distribution of Unigenes mapped to different KEGG pathways. Figure S2. Statistical results of differential expression analysis. The *x*-axis represents the comparison groups for differential analysis, and the *y*-axis shows the number of differentially expressed genes. In the color scheme, red indicates upregulated genes, while green represents downregulated genes. Table S1. Differential metabolites. Table S2. Overall statistics of the sequence. Table S3. Summary of annotation results. Table S4. Statistical results of differential expression analysis. Table S5. SRA accession numbers for transcriptomics analyses. Data S1. Secondary metabolites identified by untargeted metabolomics.
