# Supplementary material for: Utilizing Microbial Inoculants to Alleviate Continuous Cropping Obstacles: Insights into the Metabolites and Transcriptomic Responses of Pinellia ternata
Source: Metabolites. 2025 Mar 11;15(3):189. doi: 10.3390/metabo15030189 (PMC11943613; doi:10.3390/metabo15030189)
Supplement: Supplementary file 1 [file metabolites-15-00189-s001.zip › metabolites-3445635-supplementary.pdf]

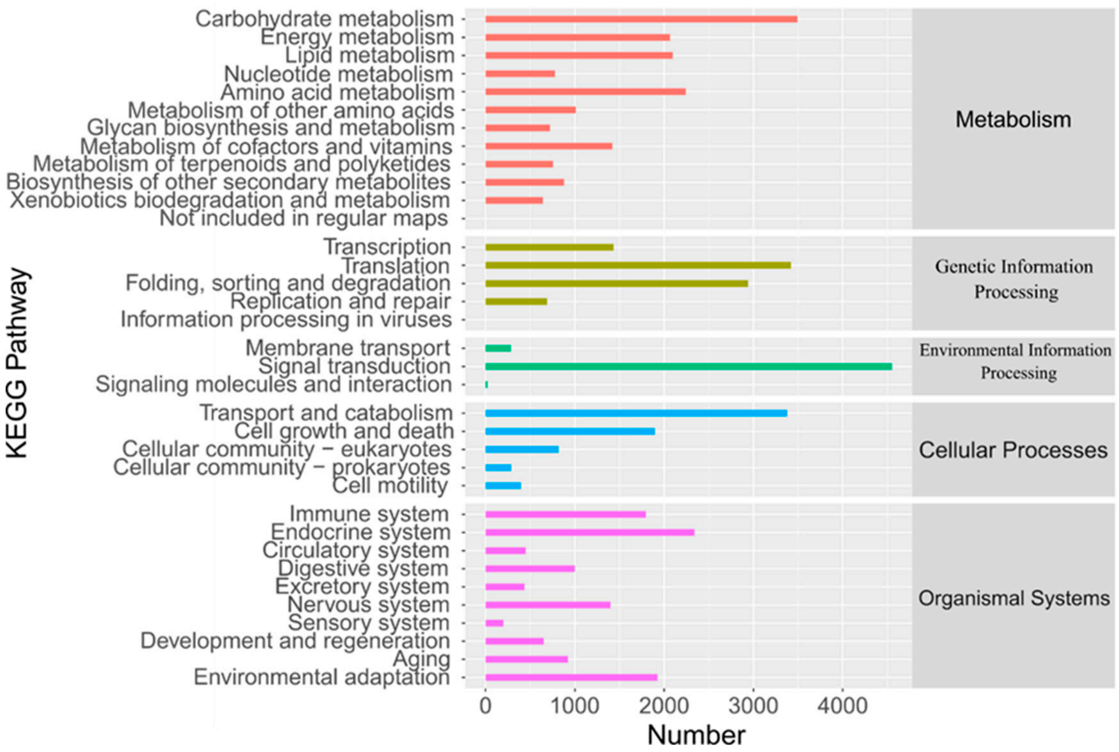

Figure S1. KEGG Annotation Statistics.Statistical results of the KEGG Pathway annotation, showing the distribution of Unigenes mapped to different KEGG pathways.

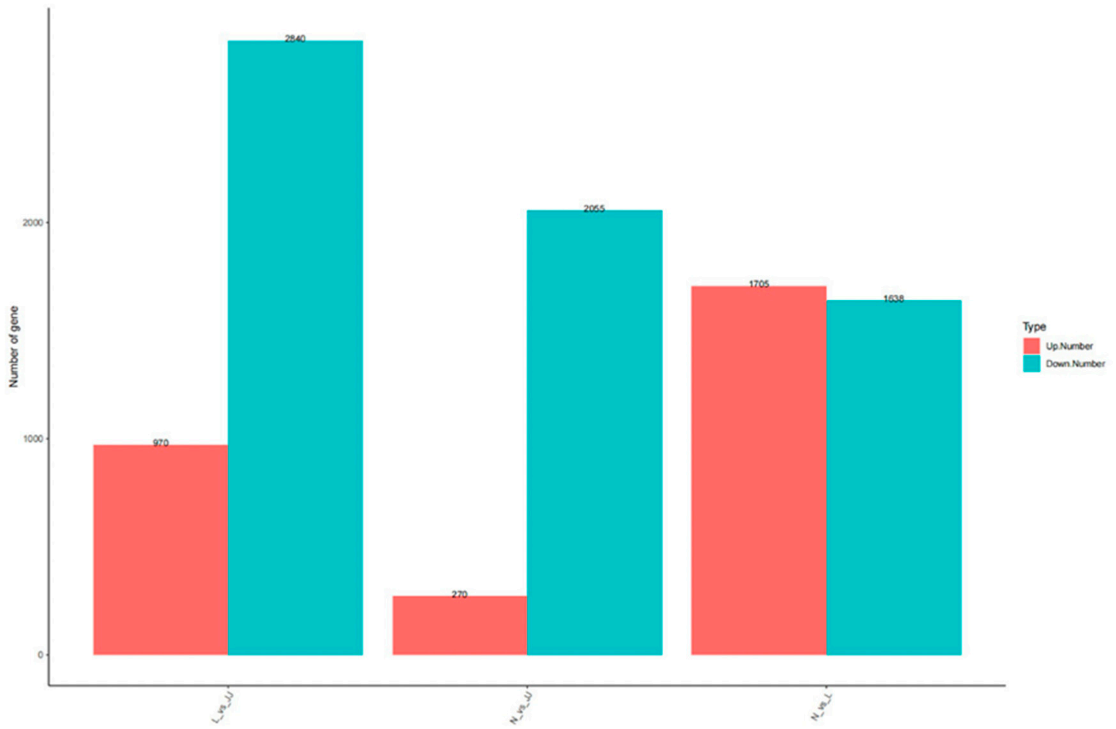

Figure S2. Statistical Results of Differential Expression Analysis. The x-axis represents the comparison groups for differential analysis, and the y-axis shows the number of differentially expressed genes. In the color scheme, red indicates upregulated genes, while green represents downregulated genes.

Table S1. Differential Metabolites

ID	Name
M1000.pos	LysoPE(0:0/18:0)
M1019.pos	LysoPE(20:2(11Z,14Z)/0:0)
M1051.pos	Zearalenone
M269.pos	Fraxinellone
M396.pos	(E)-3,10-Dihydroxy-4,9-dimethyldodec-6-enedioic acid
M492.pos	3-Hydroxy-cis-5-octenoylcarnitine
M662.pos	Lanceolatin B
M783.pos	Helinorbisabone
M172.neg	Isocitric acid
M535.neg	Aegineoside
M92.neg	Citric acid
M194.neg	Androsin
M315.neg	Mellein
M857.neg	L-Erythrulose
M876.neg	Benzyl 6-O-(4-carboxy-3-hydroxy-3-methylbutanoyl)-.beta.-D-glucopyranoside
M914.neg	Clemastanin B
M1205.pos	9,10-Dihydroxystearate
M1211.pos	trans-Cinnamate
M208.pos	Glucose
M212.pos	Sorbose
M213.pos	Galactose
M214.pos	Tagatose
M215.pos	Fructose
M216.pos	Allose
M59.pos	Apigenin-7-O-rutinoside
M70.pos	Rhoifolin
M804.pos	Fusarin_C
M968.pos	cynarin
M979.pos	2,4-dihydroxyheptadec-16-enyl acetate
M996.pos	N-Nonanoyl-L-homoserine lactone
M1064.pos	Neogrifolin
M32.pos	(卤)-Dihydroactinidiolide
M832.neg	epiyangambin
M974.neg	ROTENONE
M940.neg	Daphylloside
M1139.pos	Hericerin
M327.pos	Apigenin 7-glucoside

M789.pos	Allyl_isothiocyanate
M1094.neg	20-Oxopregn-16-en-3-yl 6-deoxy-.alpha.-L-mannopyranosyl-(1->4)-[.beta.-D-glucopyranosyl-(1->2)]-.beta.-D-glucopyranoside
M311.pos	Nootkatone

Table S2. Overall Statistics of the Sequence

	Transcript	Unigene
Total Length(bp)	588074418	165324345
Sequence Number	598893	195693
Max. Length (bp)	15592	15592
Mean Length (bp)	982	845
N50 (bp)	1346	1096
N50 Sequence No.	126017	39907
N90 (bp)	439	388
N90 Sequence No.	437702	147215
GC%	43	41

Table S3. Summary of Annotation Results

Database	Number	Percentage
NR	79950	36.37
GO	44538	20.26
KEGG	37316	16.97
Pfam	52731	23.99
eggNOG	81293	36.98
Swissprot	64172	29.19
In all database	16108	7.33

Table S4. Statistical Results of Differential Expression Analysis.

Treatments	Up Number	Down Number	DEG Number
JJ	970	2840	3810
L	270	2055	2325
N	1705	1638	3343

Summary of the differentially expressed genes between groups, including fold change and significance values. Treat: Experimental group samples; Control: Control group samples; Up-regulated Genes: Genes that are upregulated in the experimental group (Treat) compared to the control group (Control); Down-regulated Genes: Genes that are downregulated in the experimental group (Treat) compared to the control group (Control); Total DEGs: Total number of differentially expressed genes between the experimental

group (Treat) and the control group (Control).

Table S5. SRA accession numbers for transcriptomics analyses

Treatments	SRA accessions
JJ1	SRR32376063
JJ2	SRR32376062
JJ3	SRR32376061
L1	SRR32376060
L2	SRR32376059
L3	SRR32376058
N1	SRR32376057
N2	SRR32376056
N3	SRR32376055
